# Task-Related Edge Density (TED)—A New Method for Revealing Dynamic Network Formation in fMRI Data of the Human Brain

**DOI:** 10.1371/journal.pone.0158185

**Published:** 2016-06-24

**Authors:** Gabriele Lohmann, Johannes Stelzer, Verena Zuber, Tilo Buschmann, Daniel Margulies, Andreas Bartels, Klaus Scheffler

**Affiliations:** 1 University Hospital, Department of Biomedical Magnetic Resonance Imaging, University of Tübingen, Tübingen, Germany; 2 Magnetic Resonance Centre, Max-Planck-Institute for Biological Cybernetics, Tübingen, Germany; 3 Fraunhofer Institute for Cell Therapy and Immunology, Leipzig, Germany; 4 European Molecular Biology Laboratory, European Bioinformatics Institute, Wellcome Trust Genome Campus, Hinxton, Cambridge, United Kingdom; 5 Max-Planck-Institute for Human Cognitive and Brain Sciences, Leipzig, Germany; 6 CIN Vision & Cognition Group, Centre for Integrative Neuroscience, Tübingen, Germany; University of Texas at Austin, UNITED STATES

## Abstract

The formation of transient networks in response to external stimuli or as a reflection of internal cognitive processes is a hallmark of human brain function. However, its identification in fMRI data of the human brain is notoriously difficult. Here we propose a new method of fMRI data analysis that tackles this problem by considering large-scale, task-related synchronisation networks. Networks consist of nodes and edges connecting them, where nodes correspond to voxels in fMRI data, and the weight of an edge is determined via task-related changes in dynamic synchronisation between their respective times series. Based on these definitions, we developed a new data analysis algorithm that identifies edges that show differing levels of synchrony between two distinct task conditions and that occur in dense packs with similar characteristics. Hence, we call this approach “Task-related Edge Density” (TED). TED proved to be a very strong marker for dynamic network formation that easily lends itself to statistical analysis using large scale statistical inference. A major advantage of TED compared to other methods is that it does not depend on any specific hemodynamic response model, and it also does not require a presegmentation of the data for dimensionality reduction as it can handle large networks consisting of tens of thousands of voxels. We applied TED to fMRI data of a fingertapping and an emotion processing task provided by the Human Connectome Project. TED revealed network-based involvement of a large number of brain areas that evaded detection using traditional GLM-based analysis. We show that our proposed method provides an entirely new window into the immense complexity of human brain function.

## 1 Introduction

The human brain is a large-scale network consisting of approximately 85 billion neurons that form a vast number of subnetworks on all spatial scales [1]. The intrinsic connectivity within and across those networks enables the coexistence between local processing of information in specialised circuits and large-scale integrative processes, involving multiple remote sites. The network connectivity features organization principles such as small-worldness [2]) and it is likely that this architecture itself is crucial for the rich dynamic repertoire of flexibly accessible brain functions [3–5].

Traditionally, brain mapping techniques using functional magnetic resonance imaging (fMRI) have focused on studying brain areas separately in a voxel-by-voxel fashion. The key idea behind such univariate approaches is to identify task- or stimulus-related changes of the blood-oxygen-level dependent (BOLD) signal activity on the local level. The most prominent example is statistical parametric mapping using the general linear model [6, 7]. Within a univariate framework integrative processes and the functional interplay between remote brain regions generally remain inaccessible because voxels are treated *independently* from each other [8–11]. For this reason, the neuroimaging community is shifting away from this rather segregationist to a more integrative perspective of brain function [9, 11–17], such as network based approaches. In the following, we give a brief overview over existing methods that go beyond traditional GLM-based activation maps.

Seed-based approaches investigate how the statistical dependency between a seed voxel or area changes with respect to the rest of the brain. The most prominent examples are correlation-based approaches [18], where the correlation between the time series of the seed area to all other voxels is computed. A widely used method is the *psycho-physiological interaction* (PPI) method [19] and its generalisation [20], where the interaction usually is computed after deconvolution of the fMRI signal into the neural space [21].

**Fig 1:**
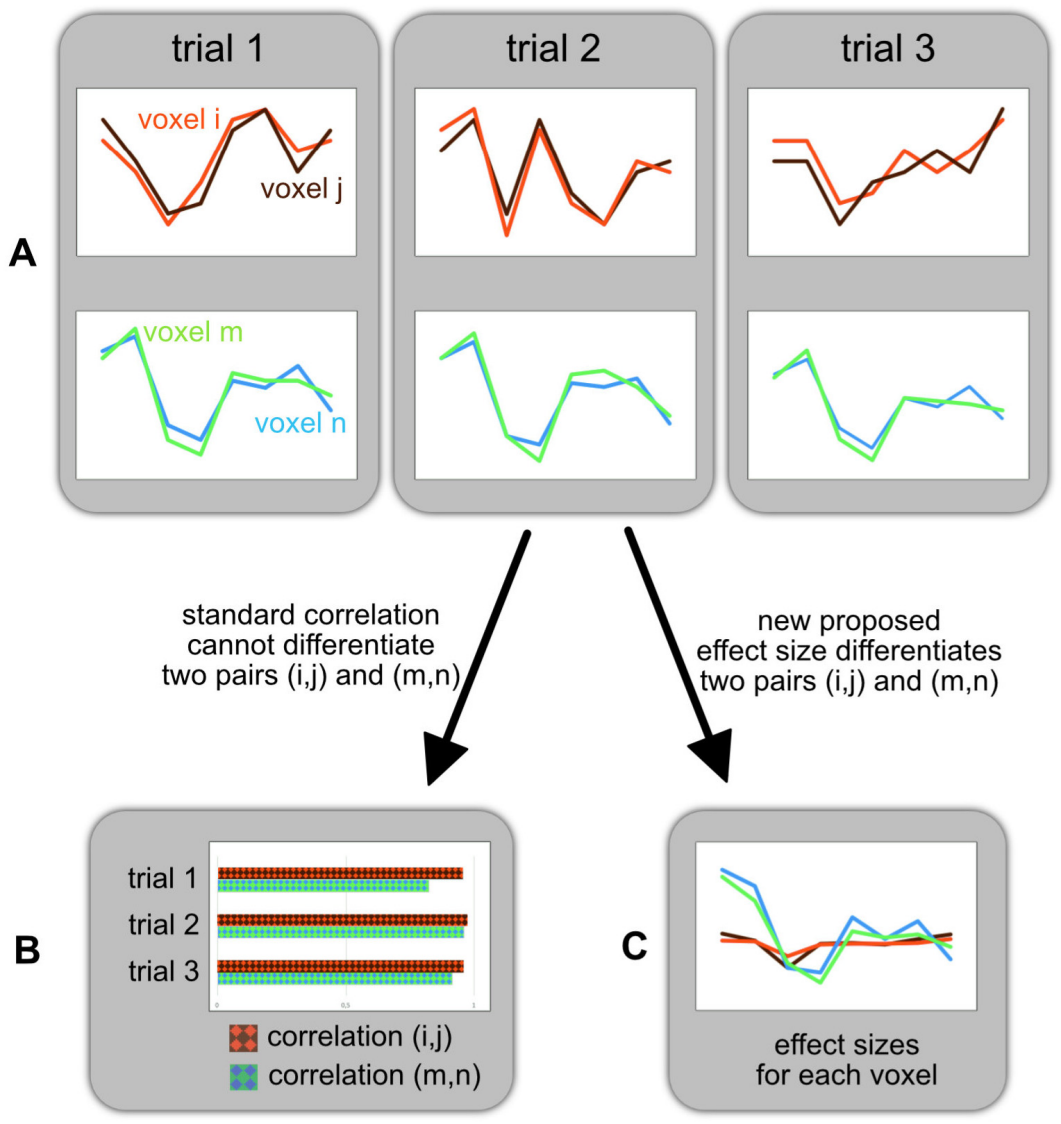
Illustration of a potential problem in correlation-based statistics. (A) Hypothetical time courses of two pairs of voxels (*i*, *j*) and (*m*, *n*) in three experimental trials of the same condition are shown here. It is clearly visible that the voxel pair (*i*, *j*) has a low inter-trial consistency, while the pair (*m*, *n*) has a high one. (B) In standard correlation-based statistics, the correlation between pairs of voxels is computed for each trial. Here, the *correlations* between (*i*, *j*) are consistent across trials, but their temporal profiles are not. In correlation-based statistics (CBS), only the consistency of correlation values is considered while the consistency of the temporal profiles within a trial is ignored. Therefore, in CBS one might erroneously conclude that voxels *i*, *j* belong to the same task-related network. The voxel pair (*m*, *n*) on the other hand also shows consistent temporal profiles and is therefore more likely to belong to the same network. (C) We propose a new measure of synchronization based on effect sizes, taking into account the inter-trial consistency. Our measure is able to separate between the voxel pairs (*i*, *j*) and (*m*, *n*); the voxel pair with low inter-trial consistency receives low scores.

The weak point of seed-based methods is their inability to reveal *global* changes of functional reorganisation. Only differences *relative to the seed area* can be depicted so that just a small part of the picture is revealed. Thus, a full exploration would require a multitude of seed-based analyses (i.e. one for each grey matter location) and to combine the resulting maps in a second step. It is easy to see that such a procedure constitutes a daunting multiple comparisons problem, which ultimately renders a whole-brain approach infeasible. The choice of the seed region itself may also be problematic especially if solely derived from GLM-based activation maps [22]. A further problem arises from analysing *correlations* in time series, as differences in correlations are in general not very reliable indicators of membership in a network. Indeed, two voxels may show strong correlations over many trials of the same experimental condition, and yet their time courses may differ widely from trial to trial, so that task-related network membership cannot be deduced from correlations alone, see Fig 1.

An alternative way of performing network-based analyses is to use parcellation schemes, reducing the number of network nodes. For instance, *condition-specific networks* [23] reveals changes in the whole-brain connectivity structure that occur as response to a task, depicting the variation of functional connectivity between pairs of regions. Similarly, *network-based statistics* [24] evaluates changes in the network structure and incorporates a solution to the multiple comparisons problem based on a graph-based connected components methodology. Thus, this method principally allows a larger number of smaller regions. Further methods include the adaptation of PPI on parcellations [22], which enables the investigation of global changes. At the other end of the spectrum in terms of involved brain regions stands *dynamic causal modelling (DCM)*, which attempts to investigate causal influences within very small networks [25]. DCM’s validity was challenged in [26].

Parcellating the brain into regions comes with a number of issues, however. Existing parcellation schemes are generally not based on anatomical features such as cyto- or myeloarchitecture because such features are very difficult to detect with current MRI acquisition and segmentation techniques [27]. As a consequence, parcellations may often give rise to nonlinear properties, where small deviations in the size of regions can result in large changes in underlying network connectivity [28]. Therefore it is no surprise that the choice of parcellation scheme and thus the number of regions have a substantial impact on the resulting network metrics [29–32]. Furthermore, region-based approaches assume functional homogeneity within the regions [33]. This is particularly troubling if the regions are large enough so that they can be further subdivided into parts that feature heterogeneous connectivity profiles (e.g. see [34–38]). Averaging within such heterogeneous regions may effectively hamper the detection of subtle connectivity changes that occur only in a subregion. Furthermore, any type of parcellation implies that it is not possible to quantify the total number of connections between regions [39]. Therefore, it has been suggested that voxel-level approaches in the context of network analysis are preferable [33].

Several other algorithms target only network hubs rather than entire networks, e.g. [40, 41] and are therefore not comparable to the present approach. Other methods such as multivariate pattern analysis (MVPA) [42] or independent component analysis (ICA) [43] also fall into a different domain, and are therefore not discussed here.

The publications listed above all contributed immensely towards a network-based understanding of brain function. However, they all suffer from some limitations, e.g. they require a presegmentation of the data, or they do not offer a mechanism for statistical inference, or they depend on a particular hemodynamic model. The dependence on a hemodynamic model was found to be problematic in a recent study by Gonzalez-Castillo et al. who tested a range of different hemodynamic response models and found wide-spread activations which had previously evaded detection [44]. They ascribed the sparsity of classical activation maps to high noise levels and overly strict response models.

Therefore, our goal in this paper is to establish a new method for fMRI data analysis that fulfills the following requirements. It should

1. identify task-related changes in network configuration,
2. not require any presegmentations,
3. be free from any specific hemodynamic response model,
4. and incorporate rigorous statistical inference.

To achieve this goal, we characterise functional networks as large-scale, task-related *collective synchronisations* of the BOLD signal measured at voxel-level resolution. At the heart of our method is the concept of spatially localised and task-related edge density motivating us to call this algorithm “TED” (Task-related Edge Density). In short, TED identifies edges in a brain network that differentially respond in unison to a task onset and that occur in dense packs with similar characteristics. We found TED to be a very strong marker for dynamic network formation that easily lends itself to statistical analysis using large scale statistical approaches.

In the following, we describe our proposed algorithm and demonstrate its applicability for dynamic network discovery in task-based fMRI data provided by the Human Connectome Project (HCP) [45].

## 2 Materials and Methods

Networks consist of nodes which are interconnected by edges. We define nodes as voxels and the weight of an edge between any pair of voxels as *task-related changes in dynamic synchronisation* between their respective times series.

Our algorithm supports experiments presented in a block design with non-overlapping trials of sufficient length to allow for a connectivity analysis. It computes a change in synchronization between two experimental conditions and it requires several repetitions of trials of both conditions to permit a valid statistical inference. Note that our method strictly requires such a differential between two conditions, as we derive our null model by random permutations of the task label. Both conditions should be implemented in the same trial structure and should both have a definite trial onset.

The algorithm proceeds in six steps. First, the data are preprocessed using a standard preprocessing pipeline which must include a correction for baseline drifts. Second, we define a measure *z* of task-related differential synchronisation for each edge in the network. In a third step, the *z*-values are normalised. Fourth, a measure called “local edge density” (*D*_*e*_) is computed for each edge, after which local edge densities are subjected to a statistical inference procedure to assess which edges are significantly affected by the experimental task. Finally, we propose methods for visualising the results. In the following, we will describe the six processing steps of TED in more detail.

### Step 1. Preprocessing

The TED algorithm assumes that the fMRI data have been preprocessed using some standard preprocessing pipeline. This should generally include corrections for motion, slicetiming, and EPI-related distortions as well as a removal of baseline drifts. In the case of multi-subject studies, a geometric alignment with the MNI anatomical template is needed. Physiological noise removal should be included into the preprocessing chain if there is reason to assume that it differentially affects the two task conditions.

### Step 2. Obtaining a measure *z* of task-related differential synchronisation

Let *A* and *B* denote two experimental conditions such as left hand versus right hand fingertapping presented in a block design with multiple trials for each experimental condition. These trials may either come from several subjects where each subject is represented by one single trial. Alternatively, we may perform a single subject analysis with multiple trials per condition within the same subject.

For simplicity, we assume that all trials have the same duration *T*, and there are *K* number of trials per condition. For condition *A*, let $v_{i}^{A}\left(k,t\right)$ denote the time course of voxel *i* of trial *k* at time *t*. We now define a measure that quantifies the amount of task-related change in connectivity between any two voxels *i* and *j* which we call *differential synchronisation* *z*.

The concept of “differential synchronization” is designed to enforce a very relevant precondition in establishing connectivity, namely inter-trial consistency. Specifically, two voxels may show strong correlations over many trials of the same experimental condition, and yet their time courses may differ widely from trial to trial (see Fig 1). Differential synchronization penalizes such inconsistencies.

We first compute the average *μ* and standard deviations *σ* across all trials as follows.

$$\begin{array}{c} \mu_{i}^{A}\left(t\right)=\frac{1}{K}\sum_{k=1}^{K}v_{i}^{A}\left(k,t\right) \end{array}$$ (1)

$$\sigma_{i}^{A}\left(t\right)=\sqrt{\frac{1}{K-1}\sum_{k=1}^{K}{\left(v_{i}^{A}\left(k,t\right)-\mu_{i}^{A}\left(t\right)\right)}^{2}}$$ (2)

For each voxel *i* we thus obtain an effect size at time point *t*:

$$s_{i}^{A}\left(t\right)=\frac{\mu_{i}^{A}\left(t\right)}{\sigma_{i}^{A}\left(t\right)}$$

The synchronisation $\theta_{i,j}^{A}$ between voxels *i* and *j* in condition *A* is then defined as the z-transformed linear correlation between *s*_*i*_(*t*) and *s*_*j*_(*t*). More precisely, we have

$$\begin{array}{c} \theta_{i,j}^{A}=\left\{\begin{array}{cc} \frac{1}{2}\log\frac{1+r_{i,j}^{A}}{1-r_{i,j}^{A}} & \text{for}\;r_{i,j}^{A}>0\\ 0 & \text{otherwise} \end{array}\right. \end{array}$$ (3)

with $r_{i,j}^{A}$ denoting the Pearson correlation coefficient between $s_{i}^{A}$ and $s_{j}^{A}$.

The synchronisations for experimental condition *B* are computed analogously.

We now have two *n* × *n* matrices Θ_*A*_ and Θ_*B*_ each recording the task-related synchronisation in conditions *A*, *B* for all pairs of voxels *i*, *j* = 1, …*n* where *n* is the number of voxels. Based on these two matrices, we introduce a measure of differential synchronisation *z* defined as an element-wise subtraction:

$$\begin{array}{c} z_{i,j}=\theta_{i,j}^{A}-\theta_{i,j}^{B} \end{array}$$ (4)

**Fig 2:**
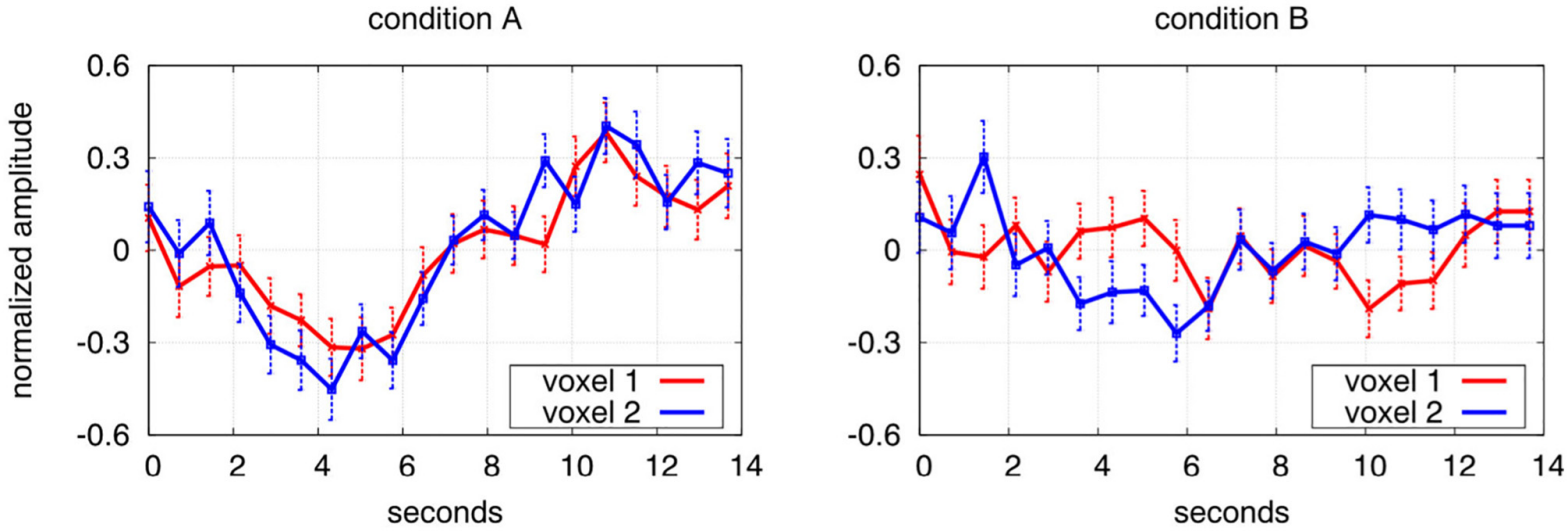
Illustration of differential synchronisation *θ*_*i*, *j*_. The figure shows mean *μ*(*t*) and standard errors *σ*(*t*) across 100 trials in a pair of voxels *i* and *j*. The left pane shows experimental condition *A*, the right pane condition *B*. The two voxels of condition *A* appear to be stronger synchronised than those of condition *B*. This is reflected by a high value of synchronisation in condition A which is $\theta_{i,j}^{A}=1.453$ while for B it is only $\theta_{i,j}^{B}=0.108$.

Note that large positive values of *z* indicate a higher synchronisation in condition *A* compared to condition *B*, see Fig 2 for an illustration.

Negative correlations are excluded in the definition of *θ* to avoid misinterpretations. Specifically, consider a case where two voxels are not correlated at all in experimental condition *A*, while showing a strong negative correlation in condition *B*. If the synchronisation *θ* were allowed to take negative values, then this would entail *z* = *θ*^*A*^ − *θ*^*B*^ ≫ 0 which might be mistaken for a task-positive involvement of the connection between these two voxels in condition *A*, even though the correlation is in fact absent. This point will be discussed in more detail later on.

### Step 3: Normalisation of *z*-values

The output of the previous step is an *n* × *n* symmetric matrix of *z*-values. Due to various confounds that may be present in the data we perform a rank-preserving transformation to the data. Such confounds may for instance be cardiac and respiratory effects or subject motion [46, 47]. Furthermore, it is well known that neuromodulatory effects (state of arousal, attentiveness, etc.) have a major impact on the fMRI signal [48]. It is extremely difficult to disentangle “true” neuronal effects from the confounding effects listed above. Therefore—instead of trying to solve this challenging problem—we propose to evade it as follows.

Specifically, we apply a rank-preserving transformation that forces the *z*-values to be Gaussian normal distributed with mean zero and standard deviation one. This can be easily achieved by any standard histogram matching algorithm [49]. We call this procedure *z*-*normalization*.

At this point, it would not make sense to apply an element-wise statistical test to the normalized *z*-values to check for significant differences from zero. The reason is that the distribution of the normalized *z*-values is identical to the theoretical null distribution which is also a standard Gaussian normal.

However, the *z*-normalization *does* preserve spatial information so that local neighbourhoods containing mostly high ranking *unnormalized* *z*-values will also have high ranking *normalized* *z*-values. The following steps aim to exploit spatial information of this type.

### Step 4: Local edge densities

**Fig 3:**
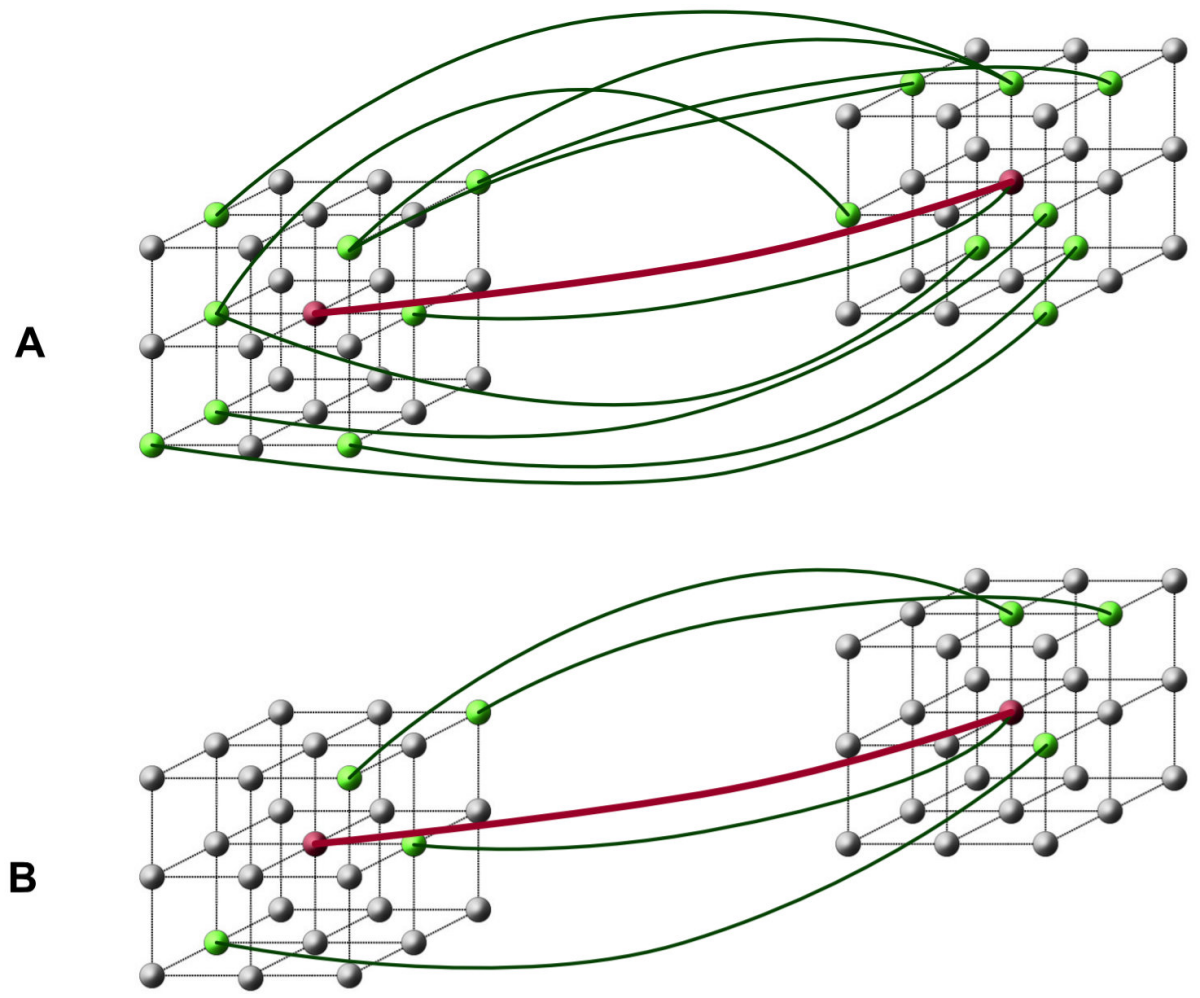
Schematic illustration of local edge density (*D*_*e*_), showing a case with high *D*_*e*_ and one with low *D*_*e*_. In this figure, voxels are depicted as small spheres. The value of *D*_*e*_ of the (thick red) edge connecting the two red voxels in the centre is computed as follows: First, we consider the 26-adjacent neighbours of its endpoints (shown here as grey and green spheres). Theoretically, the highest possible number of edges connecting any two endpoint voxels *across* the 26-neighbourhoods is 27 × 27 = 729. We define the local edge density *D*_*e*_ as the number of edges whose *z*-values are above a user-defined threshold *z*_*t*_ divided by the total number of possible edges (i.e. 729). In the above examples, lines connecting nodes (voxels) indicate supra-threshold edges. In example (A), 11 out of 729 possible edges are above threshold, thus $D_{e}=\frac{11}{729}\approx 0.015$. In example (B), only 5 out of 729 possible edges are above threshold, thus, $D_{e}=\frac{5}{729}\approx 0.007$.

We now propose a new network metric that draws on spatial adjacency as the key source of information. We call this feature “local edge density” (*D*_*e*_). The local edge density is computed for all edges in the graph that surpass an initial user-defined threshold *z*_*t*_. The value of *D*_*e*_(*i*, *j*) for an edge connecting two spatially separate voxels *i*, *j* indicates to what degree the two neighborhoods of the voxels *i* and *j* are connected with each other. A high local edge density indicates that many edges connect the two neighbourhoods, while a low local edge density indicates that only few links are present, for an illustration see Fig 3.

Quantitatively, the local edge density is defined as follows: first, the total number of possible edges between the neighborhoods of the voxels *i* and *j* is computed (omitting local connections, i.e. the start and ending point of an edge must be in different neighborhoods). Next, the number of supra-threshold edges between the neighborhoods is determined, only taking into account edges whose normalised *z*-value exceed the threshold *z*_*t*_. The fraction between this number of suprathreshold edges and the total possible number of edges then defines the local edge density *D*_*e*_. Thus, if all neighbouring edges have supra-threshold *z* values, *D*_*e*_ will be one, and if most edges fall below the threshold, *D*_*e*_ scores will approach zero. To summarize, *D*_*e*_ indicates to what degree the local neighbourhoods of pairs of voxels show a similar change of connectivity across the experimental conditions. As the local edge density is only computed for edges whose *z* is larger than *z*_*t*_, all other edges are defined to have an *D*_*e*_ of zero. In our experiments, we considered the top 1 percent of all edges so that *z*_*t*_ was set to 2.33. Note that the computation of *D*_*e*_ requires a specification of adjacency. In our experiments, we used 26-neighbourhoods, but 18- or 6-adjacencies may also be considered.

Also note that we exclude short edges from further analysis. A short edge is an edge whose endpoints *i*, *j* have a Euclidean distance of less that 15mm. The reason for doing this is that the two neighbourhoods should be non-overlapping, and because of spatial smoothness we additionally increased the minimum distance so that the borders of the two neighbourhoods are at least three voxels apart.

### Step 5: Statistical inference

In order to correct for multiple testing we employ a procedure controlling the false discovery rate (Fdr) [50–52].

The original Fdr algorithm proposed in [50] requires that data points are independent and that the null distribution is uniform. Both requirements may not be fulfilled in our case. Therefore, we use a different formulation of Fdr that is well suited for large-scale statistics involving a large number of data points with complex dependencies among them [51, 52].

The basis of this Fdr approach is the assumption of a two component mixture model for the *D*_*e*_-scores based on a null and non-null component with a cumulative distribution function (cdf) *F*_0_(*D*_*e*_) and *F*_*z*_(*D*_*e*_) respectively. Additionally, we define an a priori probability for being null by *π*_0_. Then, the observed joint density *F*_*joint*_(*D*_*e*_) is given by

$$\begin{array}{c} F_{joint}\left(D_{e}\right)=\pi_{0}F_{0}\left(D_{e}\right)+\left(1-\pi_{0}\right)F_{z}\left(D_{e}\right) \end{array}$$ (5)

The Fdr is defined as the probability of being null, or a false discovery, given a *D*_*e*_-score as large or larger than the observed one [51]:

$$\begin{array}{c} Fdr\left(D_{e}\right)=\pi_{0}\left(1-F_{0}\left(D_{e}\right)\right)/\left(1-F_{z}\left(D_{e}\right)\right). \end{array}$$ (6)

For simplicity we assume *π*_0_ = 1, which is the most conservative choice. The cdf *F*_*joint*_ is estimated from histogram counts of the local edge densities using cumulative summation. As we do not have evidence for a theoretical null distribution we rely on an empirical permutation null estimate, where the null cdf *F*_0_ is estimated by using random permutation of task labels [53]. More precisely, we randomly construct a binary permutation vector *ρ* of size *K* where each entry *k* ∈ 1, …*K* indicates whether or not the task label should be swapped in trial *k*. We used Bernoulli random trials with probability *p* = 0.5 for this purpose. This yields

$$\rho \left(k\right)=\left\{\begin{array}{cc} 0 & :\text{k-th trial original}\\ 1 & :\text{k-th trial swapped} \end{array}\right.$$

The permuted group mean amplitudes for the experimental conditions *A* and *B* for voxel *i* then are defined as

$$\mu_{i}^{{}^{\prime }A}\left(t\right)=\frac{1}{K}\sum_{k=1}^{K}\left(1-\rho (k)\right)v_{i}^{A}\left(k,t\right)+\rho \left(k\right)v_{i}^{B}\left(k,t\right)$$

$$\mu_{i}^{{}^{\prime }B}\left(t\right)=\frac{1}{K}\sum_{k=1}^{K}\rho \left(k\right)v_{i}^{A}\left(k,t\right)+\left(1-\rho (k)\right)v_{i}^{B}\left(k,t\right)$$

with the standard deviations for experimental condition *A* and *B*

$$\sigma_{i}^{{}^{\prime }A}\left(t\right)=\sqrt{\frac{1}{K}\sum_{k=1}^{K}{\left[\left(1-\rho \left(k\right)\right)v_{i}^{A}\left(k,t\right)+\rho \left(k\right)v_{i}^{B}\left(k,t\right)-\mu_{i}^{{}^{\prime }A}\left(t\right)\right]}^{2}}$$

$$\sigma_{i}^{{}^{\prime }B}\left(t\right)=\sqrt{\frac{1}{K}\sum_{k=1}^{K}{\left[\rho \left(k\right)v_{i}^{A}\left(k,t\right)+\left(1-\rho \left(k\right)\right)v_{i}^{B}\left(k,t\right)-\mu_{i}^{{}^{\prime }B}\left(t\right)\right]}^{2}}$$

In other words, for the special case of the permutation vector *ρ*(*k*) = 0, *k* = 1…*K*, the above definitions simplify to the original definitions (1) and (2).

In order to ensure that spatial smoothness is preserved we apply the same permutation vector *ρ* to all voxels in the brain mask. Therefore, differences between *F*_0_ and *F*_*z*_ cannot be attributed to the spatial correlations that are generally inherent in fMRI data. As described in more detail below, a relatively small number of permutations may suffice to converge to a stable estimation of *F*_0_, and hence of the false discovery rate.

### Step 6: Visualisation of results

The previous processing step yields a set of edges that indicate significant changes in task-related connectivity. Since the number of such edges can be very large, visualisation of results may become difficult. Here we propose two different methods.

The first method is to project edges onto a “hubness map”. A voxel in the hubness map records the number of edges for which this voxel serves as an endpoint. Voxels in which many edges accumulate may be viewed as hubs in a task-specific network, and the number of edges meeting in a voxel is a measure of the voxel’s hubness. Note however, that this measure of hubness should not be confused with activation strength as can be seen from Fig 2. Here differential synchronisation goes along with a decrease in BOLD activation rather than an increase.

The second method is to display edges as lines in a 3D rendering such that each line represents an edge that survived significance thresholding. Such renderings can become quite cluttered and therefore edges that are close to each other are bundled together to produce a clearer picture [54]. We use the software package “braingl” for this purpose [55].

### Experimental Data

We applied TED to task-based fMRI data provided by the Human Connectome Project (HCP), WU-Minn Consortium [45, 56]. Specifically, we focused on two tasks, namely the fingertapping task and the emotion processing task. In both cases, we used minimally preprocessed fMRI data of 100 participants (54 females, 46 males, ranging in age between 22 and 36 years at the beginning of HCP measurement with a mean of 27.52 years).

The preprocessing protocol is described in [57]. We reduced the number of voxels and hence the computational load by downsampling to isotropic voxels of size (3.0*mm*)^3^ from the original resolution of (2.0*mm*)^3^. We also corrected for baseline drifts using a highpass filter with a cutoff frequency of 1/90 Hz. To reduce the effect of anatomical variability across subjects, we applied spatial smoothing with a Gaussian smoothing kernel of FWHM = 5mm. Spatial smoothing is however not an integral part of the algorithm and should be omitted whenever possible, particularly for single-subject analysis [58].

We manually defined a region of interest (ROI) containing about 54,000 voxels covering the entire brain including grey and white matter, subcortical structures, CSF and the cerebellum. Furthermore, we normalised the voxel-wise time series of each trial, so that their time series have mean zero and standard deviation one.

Both experiments were acquired in two separate runs with one run using left-right phase-encoding, and the other run using right-left phase-encoding. We performed the initial three steps of TED separately for each of the two phase-encoding runs resulting in two matrices of normalised *z*-values. These two matrices were then combined via a conjunction analysis by taking the element-wise minimum of the *z*-values. For the subsequent local edge density computation we used a threshold of *z*_*t*_ = 2.33.

We applied TED as described above using random permutations to estimate the null distribution. In the first experiment, we additionally tested how many permutations were needed to achieve a stable result. The TED algorithm is implemented in C/C++ and makes use of parallel computation. With 54,000 voxels TED requires about 13 GByte of main memory, and one permutation takes about three minutes of computation time on a Linux PC using 12 parallel cores.

#### Experiment 1: Fingertapping task

The experimental design is described in Barch et al. as follows [56]. While in the scanner, participants were cued visually to tap their left or right fingers, squeeze their left or right toes, or move their tongue. Each block lasted 12 seconds (10 movements), and was preceded by a 3 second cue. Since the repetition time was 720 milliseconds, there were 16.666 volumes (time steps) per trial of which we used the initial 16.

In each of the two runs, there were 13 blocks, with 2 of tongue movements, 4 of hand movements (2 right and 2 left), 4 foot movements and three 15 s fixation blocks per run, see [56]. Here, we only used the second of the two fingertapping blocks so that we have 100 trials for each condition *A* and *B* (right hand tapping and left hand tapping) in each phase-encoding run.

We applied TED as described above initially using 1000 random permutations to estimate the null distribution. Furthermore, to investigate the minimum number of permutations that are required for stable results, we applied TED using a total of 10000 random permutations. The results are shown in section 3.

#### Experiment 2: Emotion processing task

In this experiment, participants were cued to decide which of two faces matched the face shown on top of the screen. In a second experimental condition, an analogous task was done using shapes instead of faces. The faces had either angry or fearful expressions, see [56].

Each of the two runs included three face blocks and three shape blocks where each block has a duration of 21 seconds. Here we only used the first face block, as well as the first shape block. Thus, we had 100 “face” and 100 “shape” trials in each run where each trial originated from one of the 100 subjects. We applied TED as described above using 1000 random permutations to estimate the null distribution. We computed both the “face-shape” and the reverse “shape-face” contrast. The results are discussed in the next section.

## 3 Results

### Experiment 1: fingertapping task

**Fig 4:**
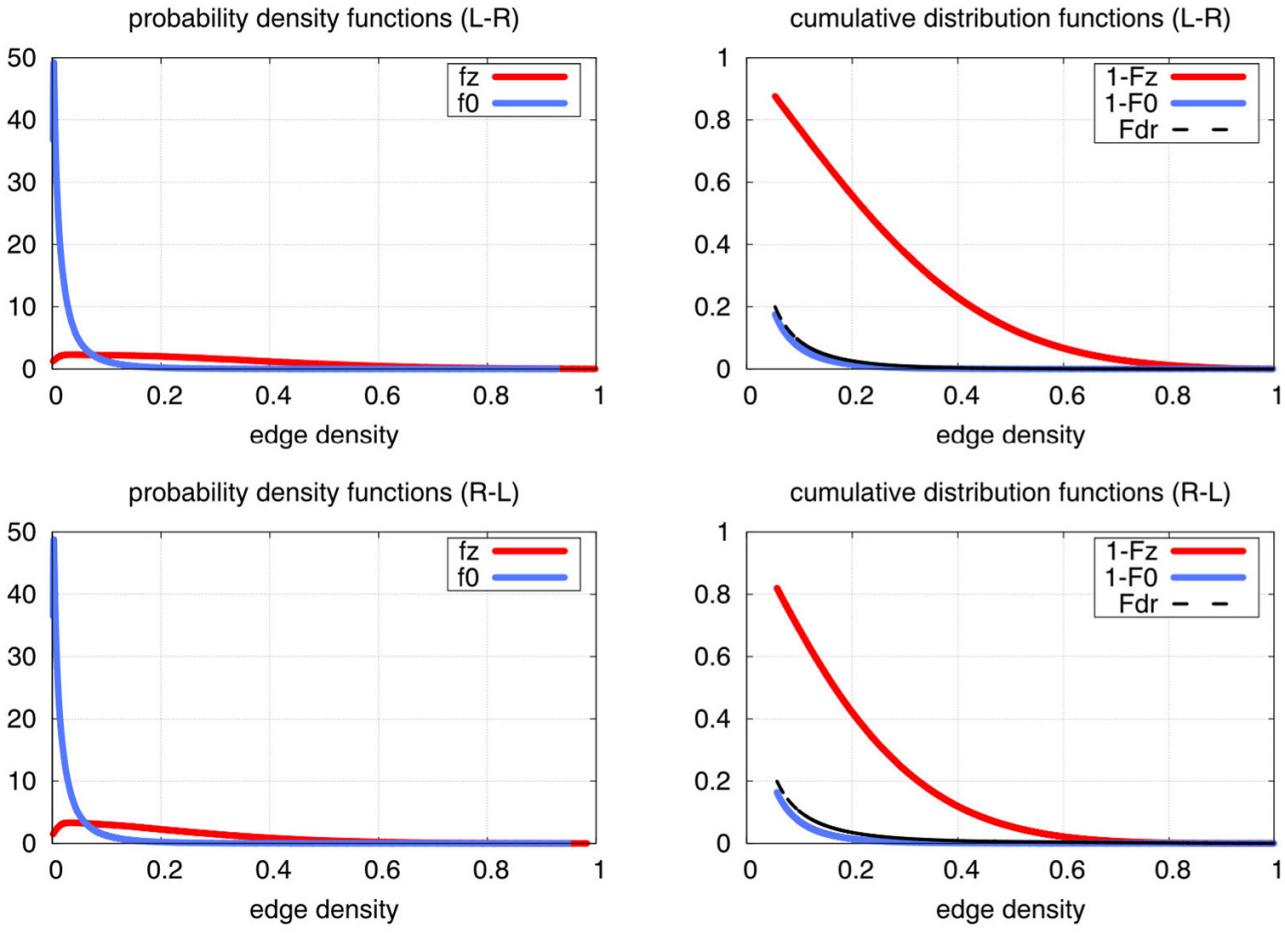
Probabilities of the local edge density estimated from fMRI fingertapping data. The top rows contrasts left hand minus right hand tapping, the bottom row is the reverse contrast (right hand minus left hand). The left plots show estimations of the probability density functions *f*_0_ (permutation-derived) and *f*_*z*_ (no permutation). The plots in the middle show the corresponding cumulative distribution functions (CDF) *F*_0_ and *F*_*z*_. Note that the differences between the null and the “real” distributions are massive. The CDFs are used to estimate the false discovery rates (Fdr) which are indicated here by dashed black lines. For better visualization, the same Fdr curves are also shown in the plots on the right. The Fdr is used to determine a significance threshold. For the left minus right contrast, the cutoff was found to be *D*_*e*_ > 0.1435, i.e. for edges with *D*_*e*_ > 0.1435 the false discovery rate falls below 0.05. For the reverse contrast, the cutoff was *D*_*e*_ > 0.1521. The estimation of *F*_0_ is based on 1000 random permutations.

We first contrasted global connectivity related changes for right hand minus left hand tapping. Fig 4 shows the distributions *F*_*z*_ and *F*_0_ that were used to estimate statistical significance. The corresponding density functions *f*_*z*_ and *f*_0_ are also shown in this figure. They were estimated using Gaussian kernels whose bandwidth was defined via Silverman’s rule [59]. At a false discovery rate of *Fdr* < 0.05 the local edge density cutoff was found to be *D*_*e*_ = 0.1521, i.e. edges with *D*_*e*_ > 0.1521 can be assumed to indicate a significantly stronger synchronisation in left hand versus right hand tapping. Corresponding plots for the reverse contrast can also be found in Fig 4. Note that far fewer than 1000 permutations would have sufficed to reach a similar result, as shown in S5 Fig.

**Fig 5:**
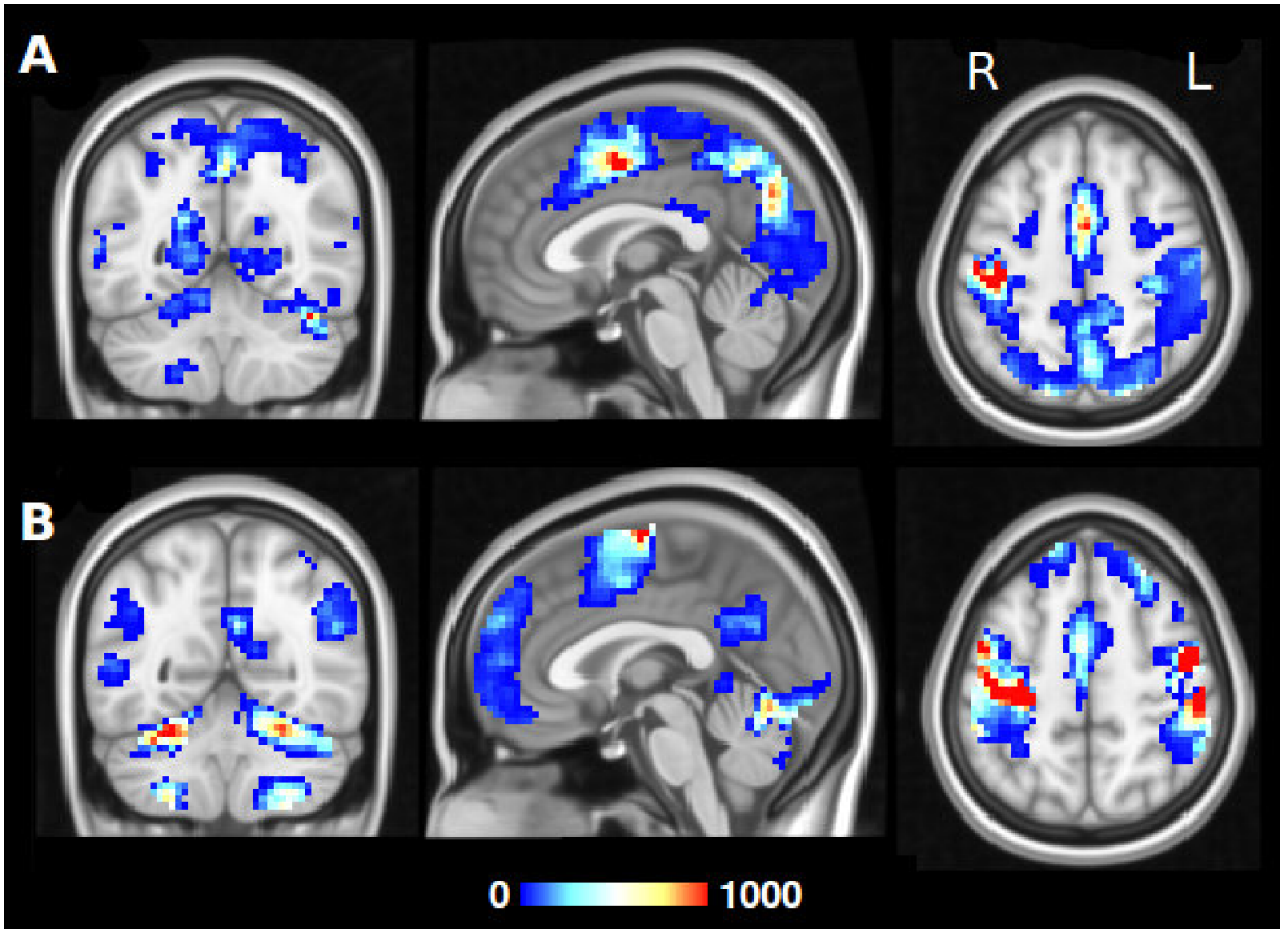
Task-dependent dynamic reconfiguration of whole-brain networks. We depict the reconfiguration using hubness maps on basis of fMRI data fingertapping data of the Human Connectome Project. The hubness maps indicate the number of network edges that feature a significant change between the two experimental conditions. The top row (A) contrasts right hand minus left hand tapping, the bottom row (B) shows the reverse contrast. The colours encode the number of edges with *Fdr* < 0.05 having one of their endpoints in the respective colour-coded voxel and ranges from 1 to 1000. This number can be interpreted as a measure of “hubness”. Thus, red values in the above figure indicate hubs where many edges accumulate in a voxel. See also S1 and S2 Figs.

Fig 5 shows a resulting hubness map produced as described above in step 6. Voxels that are colour-coded are endpoints in an edge significantly affected by the task. Voxels in which many edges accumulate may be viewed as “hubs” in a task-specific network, and the number of edges meeting in a voxel is a measure of the voxel’s “hubness”. The upper panel indicates the hubness for edges in the contrast right hand minus left hand. Tapping with the right hand as opposed to the left increased the global connectivity in supplementary motor areas, right and left motor cortex, somatosensory areas, the frontal eye fields, regions in the parietal cortex and the visual cortex. On the other hand, as shown in the lower panel, left hand tapping minus right hand tapping seemed to increase the global connectivity within the bilateral motor network, the default mode network, bilateral putamen, bilateral V5, insular cortex and regions in the cerebellum.

In the same figure it can be seen that several regions appear involved in both contrasts because they participate in different task-relevant networks depending on the experimental condition. As an example for this, we investigated the connectivity of the right primary motor cortex (hand knob region), selecting only the edges which have one of their endpoints here (S3 Fig).

Note that these results depend on an initial *z*-threshold which was set somewhat arbitrarily to the top 1-percent level. However, as shown in S6 Fig, the results appear to be fairly consistent across various other thresholds.

**Fig 6:**
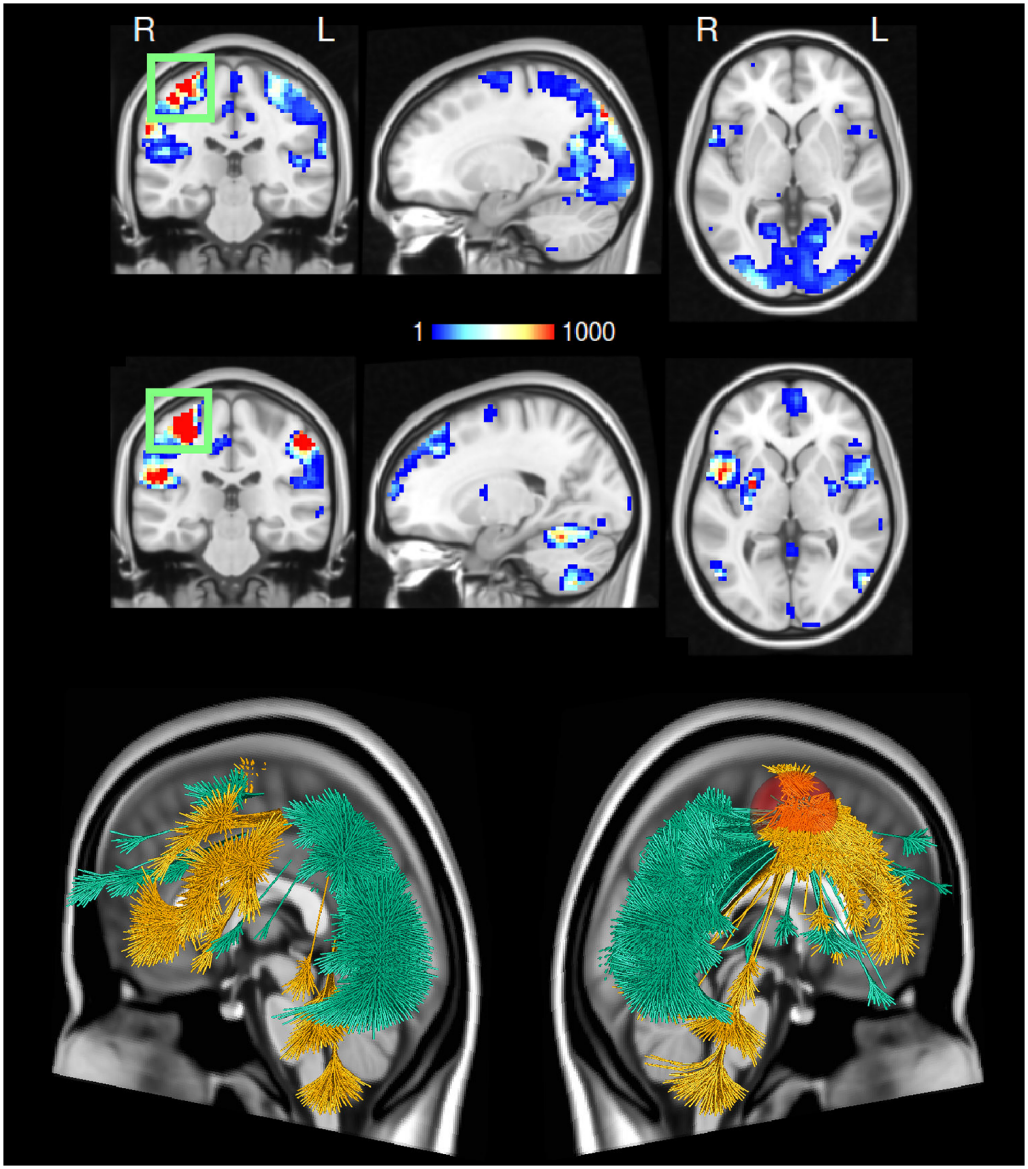
Task-related reconfiguration of the right-hemispheric primary motor cortex. In a TED analysis, a single region may appear in opposite maps because the contrast is computed between edges, not between voxels. Therefore, *edges* can only appear in one contrast whereas a *voxel* may serve as an endpoint in two separate edges, where the two edges represent opposite contrasts. So in fact, the same voxel voxel may be part of two different networks that subserve different tasks. In this figure we show the participation of the right primary cortex (hand knob area, marked with a green box) in different networks, depending on the experimental condition, see discussion section. The exact shape of the region of interest is shown in S3 Fig. In the upper panel we display voxels involving all edges featuring a stronger synchronisation with the right primary motor cortex for right hand fingertapping (as compared to left hand tapping). In the lower panel we show the voxels where the synchronisation with the same area is higher for left hand fingertapping (as opposed to right hand tapping). The maps reveal a striking difference in synchronisation of the right primary motor cortex to the rest of the brain: the right hand tapping condition involves stronger synchronisation between the right motor cortex and regions in the visual and parietal cortex. On the other hand, in the left hand condition the same area synchronizes more with bilateral areas in the cerebellum, V5, the putamen and insular cortex and furthermore the medial prefrontal cortex. Below, the same data are shown using a 3D rendering using the software package “braingl” [54, 55]. Here, the synchronisation network of the right primary motor cortex (the red sphere) in the right hand minus left hand contrast is shown in green, the reverse contrast is shown in yellow.

As shown in Fig 6 this region shows a stronger synchronisation between the right motor cortex and regions in the visual and parietal cortex during the right hand tapping condition. In the left hand condition, the same area shows higher synchrony with bilateral areas in the cerebellum, V5, the putamen and insular cortex and furthermore the medial prefrontal cortex. Thus, the hand knob region participates in different networks depending on the experimental condition (Fig 5). It should be noted that such a case of differential involvement cannot be detected in a classical GLM-based analysis.

**Fig 7:**
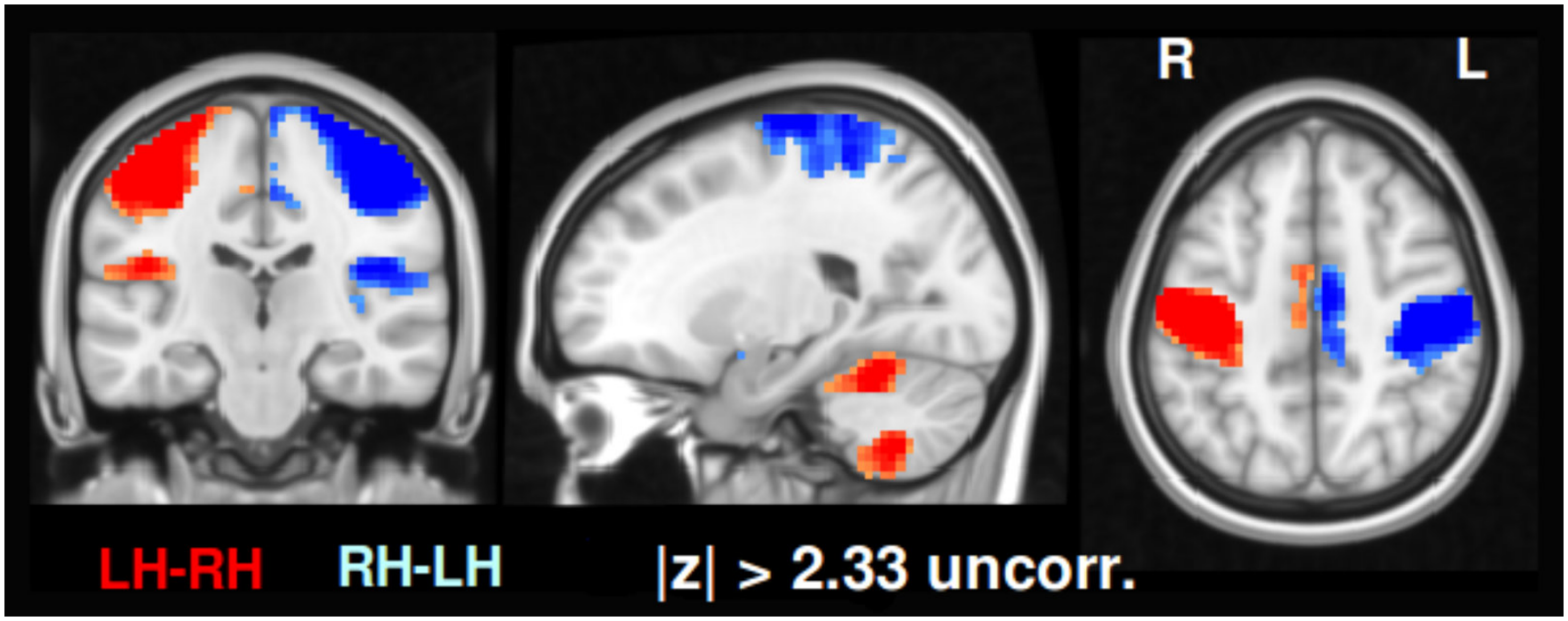
Classical univariate GLM-based analysis. For comparison to standard analysis methods, we used a univariate activation based GLM technique (see methods). We thresholded the activation map very liberally at |*z*| > 2.33 on the voxel level without correcting for multiple comparisons. See also S4 Fig.

For comparison, we performed a standard analysis using the GLM approach as implemented in Lipsia [60]. The preprocessing of the data was performed as described above. We computed activation maps for each of the two phase-encoding runs separately using the general linear model. These maps contain uncorrected *z*-values representing the contrast between left hand minus right hand fingertapping. As in the TED approach, we performed a conjunction analysis on the two maps, where the voxel-wise minimum value of both Z-maps was used for the case that both were positive, the maximum value in case both were negative, and zero for diverging signs of the *z*-values. We then thresholded the resulting conjunction map such that voxels with |*z*| > 2.33 remained. No multiple comparisons correction was applied. The resulting map contrasting left hand minus right hand fingertapping shows the voxel-wise differences in activation strength, see Fig 7. The activations include bilateral motor areas, pre-SMA, as well as the cerebellum. In comparing the TED and GLM results of Figs 5 and 7, we see that TED picks up similar regions as the GLM. However, additional hubs appear in putamen as well as in visual, parietal, medial frontal regions.

### Experiment 2: emotion processing task

We applied TED as described above using 1000 random permutations to estimate the null distribution. For the “face-shape” contrast, we obtained a local edge density cutoff *D*_*e*_ > 0., i.e. for edges with *D*_*e*_ > 0.1481 the false discovery rate falls below 0.05. For the reverse contrast, the cutoff was found to be *D*_*e*_ > 0.1493. The corresponding distribution functions are shown in S8 Fig.

**Fig 8:**
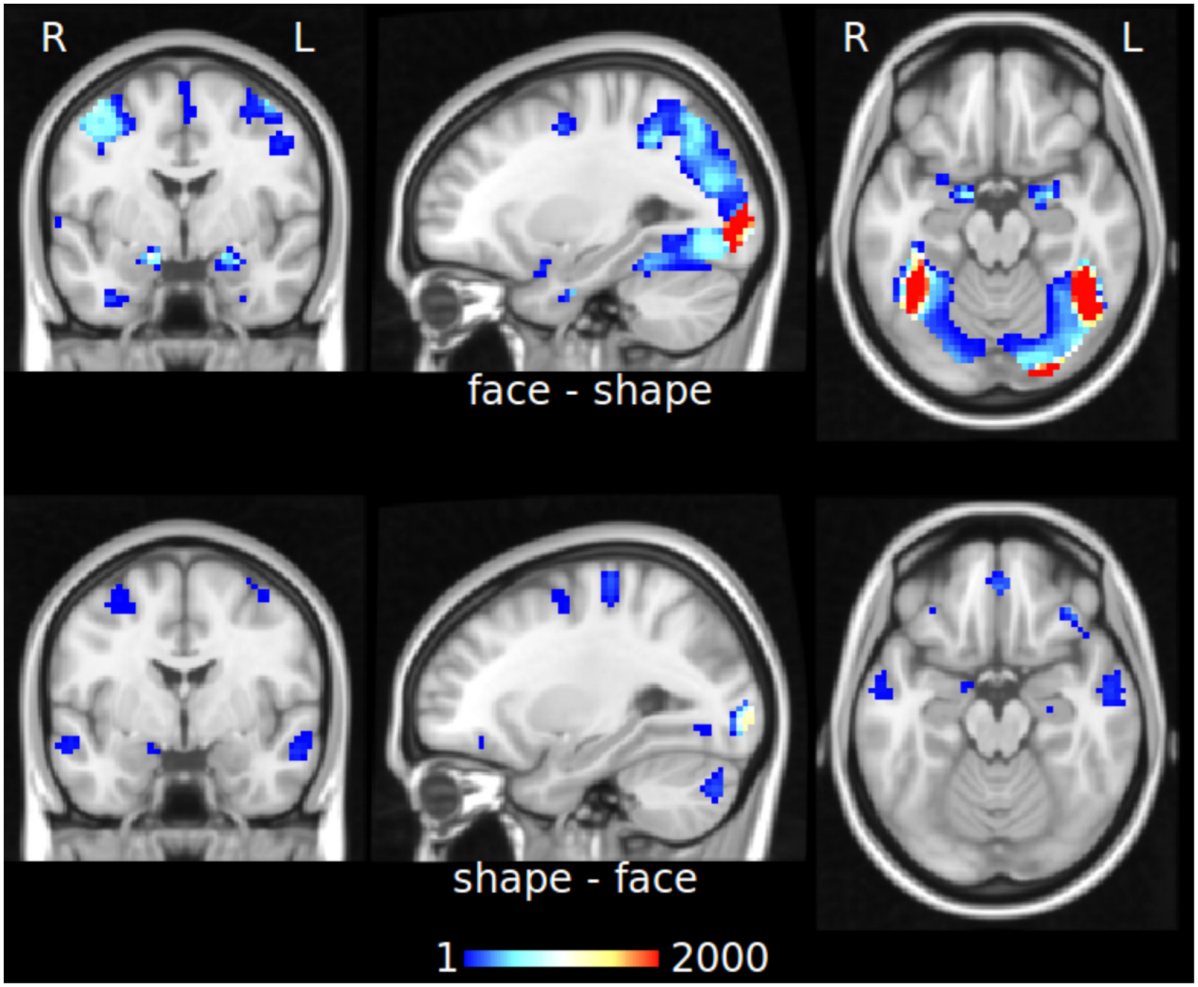
Hubness maps for the emotion processing task. The top row shows the hubness map of the “face-shape” contrast, the bottom row shows the reverse contrast. Both maps are based on TED results thresholded at *Fdr* < 0.05. Here we show slices at (x = -20, y = -6, z = 16) to facilitate comparison with the corresponding Fig 11 of Barch et al. [56]. See also S9 and S10 Figs.

Using these thresholds, we obtained a hubness map for each of the two contrasts, see Fig 8, and also S9 and S10 Figs. The “face-shape” contrast maps reveal a remarkable degree of similarity with those obtained in the original GLM results shown in Barch et al. [56], as both results involve nearly the same set of voxels. Notably though, while the GLM contrast shows a similar degree of net BOLD activity increase in the amygdala, occipital and fusiform cortices, the hubness maps reveal an overwhelmingly larger change in hubness specifically in the amygdala compared to all other regions.

## 4 Discussion

In this study, we introduced a new approach for the analysis of fMRI data called “TED” which is designed to identify task-related reconfigurations in brain networks without requiring presegmentations and without being dependent on any specific hemodynamic response function. Statistical inference was a key concern in our context because the number of statistical tests increases quadratically with the number of voxels.

At the heart of TED is the concept of “local edge density”. Two spatially remote voxels form an edge that is deemed to be task-positive if the two voxels collectively synchronise in response to the task. At the same time, such an edge is required to appear in a dense pack of neighbouring edges. This approach allowed us to make inferences at the spatial resolution of small neighbourhoods around individual voxels without requiring a presegmentation. It also freed us from the need for any explicit hemodynamic modelling.

We used a null model derived from random permutations for statistical inference. We found that the null and the real distributions of local edge densities differ massively so that statistical significance could be clearly established. The rank-preserving normalization ensures that the number of edges are the same in the null and the real model so that we may conclude that this effect is mainly driven by the spatial configurations of suprathreshold edges. The spatial structure was preserved in our random permutations so that our results cannot simply be due to the spatial smoothness that is generally inherent in fMRI data.

The results obtained with TED suggest a dominant role of local inter-connected neighbourhoods forming transient task-related networks with other local neighbourhoods. In this regard, our concept of local edge density is somewhat related to the concepts of a clustering coefficient and small-worldness [2, 61]. However, local edge density differs from the clustering coefficient in that it measures connectivity between two local neighbourhoods at spatially separate areas. In the literature, there exist several approaches comparing the differences between local and global functional connectivity profiles [62, 63]. It has been found that different brain regions exhibit a varying balance between such local and global connectivity, which may further depend on the current experimental state (i.e. task). On the other hand, the results of both Sepulcre et al. [62] and Tomasi et al. [63] indicate a strong overlap between regions which feature both increased local and global functional connectivity. Our methodology offers a potentially interesting perspective on this, as according to our interpretation global changes in connectivity may be accompanied by local interconnections, reflecting the dynamics of local subnetworks that form transient long-range connections.

TED constitutes a novel approach that complements traditional GLM based analyses in that it focuses on integration rather than just segregation of brain function. A single brain region may subserve different functions and may be invoked in response to different experimental conditions so that it can participate in different networks depending on the task. Univariate methods such as GLM fail to detect such scenarios. This is a major limitation because the brain is a complex dynamic system and large-scale interactions are the key to brain functionality [11]. TED on the other hand is especially designed to reveal such network interactions.

We presented the right-hemispheric motor area where we would expect to find effects due to handedness so that the contrast “left hand minus right hand” should not simply be the same as “right hand minus left hand” with the sign reversed. And indeed, the TED hubness maps of the first contrast show a remarkably different pattern from that of the reversed contrast—an effect that may well be ascribed to handedness [64]. Similarly, in the emotion processing task, the contrast “face—shape” revealed the amygdala as the main hub related to face-processing, in addition to the fusiform gyrus with parts of LOC. The reverse contrast no longer included the amygdala, but again fusiform and LOC regions. These maps hence reflect that faces and objects both involve LOC, yet in different ways, a result otherwise only obtainable using non-specific scramble stimuli or multi-variate pattern classifier approaches [65].

In the present study we applied TED to group level data so that spatial accuracy was limited. This was even further reduced because we had to downsample the data to (3*mm*)^3^ resolution to ease the computational burden, see [58] for a discussion on problems relating to spatial inaccuracies. These limitations are not implicit in the TED algorithm, so that it is quite possible to apply TED to single subject data at very high spatial resolutions provided sufficient computational resources exist and a sufficient number of experimental trials are acquired to yield sufficient statistics. Since TED specifically targets local neighbourhoods, we expect that the results will benefit from more precise spatial information, e.g. provided by ultra high-field MRI scanners.

Note that our present implementation of TED assumes that inter-trial variance is the only source of variation in the data. In fact, the concept of differential synchronization is based on the idea that trials belonging to the same experimental task elicit a consistent response throughout the experiment [66]. However, this assumption may not always hold true, as there are various sources of trial-to-trial variability, such as fluctuating attention [67, 68], errors in the subject’s responses [69], differences in cognitive strategy (e.g. learning), effects of age or pathology [70, 71] and ongoing spontaneous background activity such as adaptation processes [72]. As all of the above sources introduce inter-trial variance, the power of our proposed method might be reduced. This may result in false negative results, but not likely in false positives. We therefore applied TED to a more complex experimental task—the HCP emotion task—so assess whether TED would then become so conservative that it would no longer be feasible. The results however show that TED works well even in such more complex situations. Furthermore, in typical fMRI studies both inter-subject as well as intra-subject variation may be present, for instance when multiple trials are acquired of multiple subjects. One possible solution might be to concatenate the trials belonging to the same subject so that every subject is represented by one single concatenated list of trials. The implementation of this idea will be the object of future work.

In principle, our method can also be applied to resting state studies (rs-fMRI). However, the concept of differential synchronization (step 2) is then not applicable because in rs-fMRI we do not have task-related onset times that are needed to assess the synchronicity of trials. Hence, in rs-fMRI, step 2 of the algorithm will have to be replaced by a method in which correlation matrices are computed for all trials. The *z*-matrix then results from a *t*-test across these matrices. The remaining steps 3 to 6 of the algorithm can be used without any changes. Another point to consider is that our method requires input from two samples, e.g. a patient group versus a control group. It cannot be used on a single sample of resting state data because the mechanism for constructing the null model would then become invalid.

Negative correlations were excluded in our current implementation to avoid ascribing positive task involvement to negative correlations, see Eq 3. The nature of negative correlations is ambiguous as they can either result from a phase-lagged connectivity between two brain regions, or alternatively indicate truly anti-correlated networks. As this issue cannot be adequately resolved on the basis of fMRI measurements alone, we decided to avoid this issue here. Nonetheless it is clear that further research on the nature and origin of negative correlations is needed. We expect that multi-modal measurements might give more insights into this critical issue, for instance using simultaneous acquisition of large-scale electrophysiological data and BOLD fMRI.

Note that our method depends two free parameters. First, it needs an initial threshold *z*_*t*_, and second it depends on the choice of a neighbourhood definition. While the initial threshold has noticeable influence on the extent of task-positive involvement, the second parameter is much less influential, see S6 and S7 Figs.

In sum, the fact that we now have to deal with entire networks rather than univariate regions adds another level of complexity to data interpretation and visualisation. On the other hand, this complexity likely reflects much more closely the true complexity of human brain function.

## Supporting Information

S1 Fig Task-dependent dynamic reconfiguration of whole-brain networks. This map is based on the same data as Fig 5 of the main manuscript. It shows the hubness map of the contrast left hand minus right hand fingertapping.
(TIF)

S2 Fig Task-dependent dynamic reconfiguration of whole-brain networks. This map is based on the same data as Fig 5 of the main manuscript. It shows the hubness map of the contrast right hand minus left hand fingertapping.
(TIF)

S3 Fig Region of interest in the right motor area. The map shows the region of interest in the right motor area used in Fig 6 of the main manuscript.
(TIF)

S4 Fig GLM-based activation map of the fingertapping task. This map is based on the same data as Fig 7 of the main manuscript.
(TIF)

S5 Fig Impact of the number of permutations that are used to estimate the null distribution. The plot shows the distribution of the cutoff-parameter *D*_*e*_ based in dependency of the number of permutations that are used. The permutations were computed in blocks of 50, which we concatenated to investigate the stability of the cutoff local edge density *D*_*e*_. More precisely, we computed the the distribution of *D*_*e*_ based on 50, 100, 150 and 200 permutations, using 40 random draws of samples each (without replacement). The error bars indicate the standard error across the 40 draws that were performed for each number of permutation. The estimate of *D*_*e*_ clearly converges for larger number of permutations, as the mean and standard deviation converges after 100 permutations.
(PDF)

S6 Fig Impact of the initial *z*_*t*_-threshold on hubness maps. We compared three different initial *z*_*t*_-thresholds for the HCP motor task. The resulting hubness maps were binarized and overlayed over each others for the sake of comparability. The regions displayed in red are only found for the lowest (i.e. most permissive) threshold, regions in yellow are found for both the lowest and middle threshold, and finally regions in white are found in the hubness maps of all thresholds. Note that the hubness maps for the more stringent thresholds did not contain voxels that were undetected in the lower thresholds, that is, the detected regions were always a subset of the next lower threshold.
(PNG)

S7 Fig Impact of the neighborhood scheme on hubness maps. We compared three different definitions of neighborhood adjacency (26, 18 and 6) with each others. We overlayed the results in an additive manner, as in a Venn diagram: voxels in white color are found for all neighborhood schemes, pure colored voxels (red, green and blue) only for one respective neighborhood scheme (26, 18 and 6) and mixed colors (yellow, cyan, magenta) for voxels found in two schemes (26/18, 18/6, 6/26).
(PNG)

S8 Fig Probabilities of the local edge density estimated from emotion processing task. For the “face-shape” contrast, the cutoff was found to be *D*_*e*_ > 0.1481, i.e. for edges with *D*_*e*_ > 0.1481 the false discovery rate falls below 0.05. For the reverse contrast, the cutoff was *D*_*e*_ > 0.1493. The estimation of *F*_0_ is based on 1000 random permutations.
(PNG)

S9 Fig Hubness map of the emotion processing task, “face-shape” contrast. The map is based on TED results thresholded at *D*_*e*_ > 0.1481 so that *fdr* < 0.05. It is based on the same data as that of Fig 8 of the main manuscript.
(TIF)

S10 Fig Hubness map of the emotion processing task, “shape-face” contrast. The map is based on TED results thresholded at *D*_*e*_ > 0.1493 so that *fdr* < 0.05. It is based on the same data as that of Fig 8 of the main manuscript.
(TIF)

S11 Fig GLM-based activation map of the emotion processing task. The map shows uncorrected *z*-values thresholded at |*z*| > 2.33 following a conjunction of a GLM analysis of the two phase-encoding runs.
(TIF)
