# Supplementary figures and images for: Task-Related Edge Density (TED)—A New Method for Revealing Dynamic Network Formation in fMRI Data of the Human Brain
Source: PLoS One. 2016 Jun 24;11(6):e0158185. doi: 10.1371/journal.pone.0158185 (PMC4920409; doi:10.1371/journal.pone.0158185)

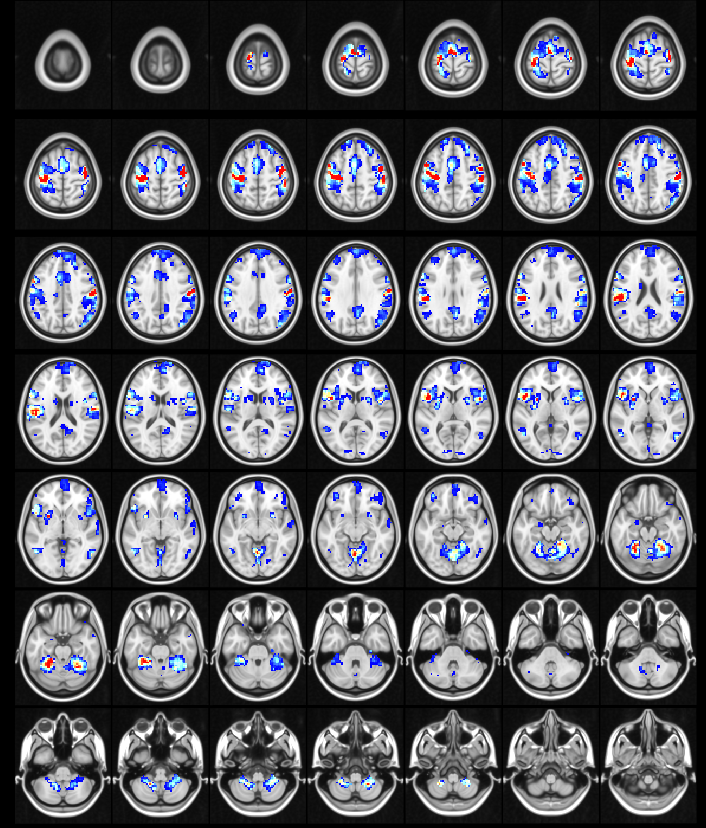

Supplement: S1 Fig — This map is based on the same data as Fig 5 of the main manuscript. It shows the hubness map of the contrast left hand minus right hand fingertapping. (TIF) [file pone.0158185.s001.tif]

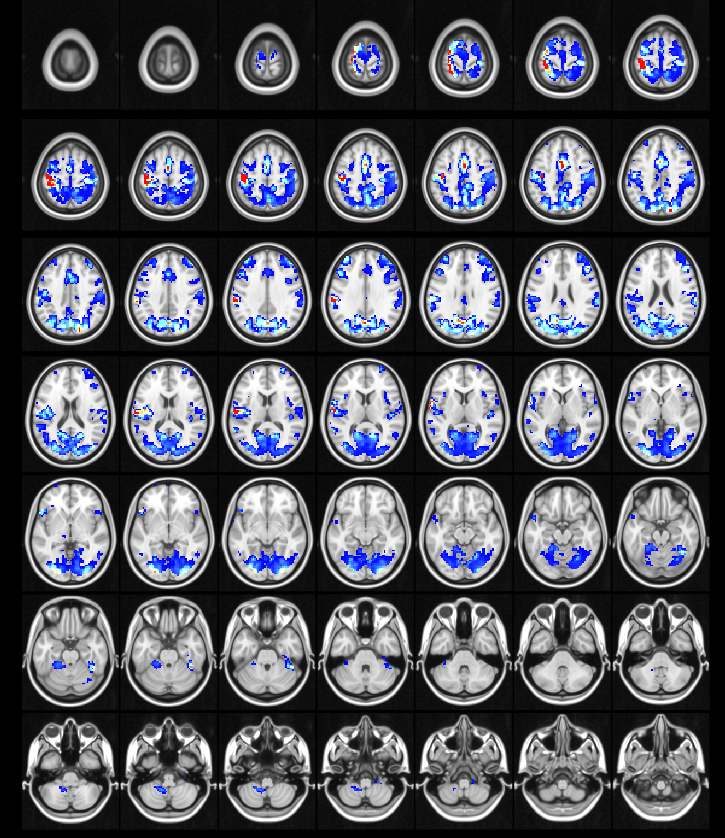

Supplement: S2 Fig — This map is based on the same data as Fig 5 of the main manuscript. It shows the hubness map of the contrast right hand minus left hand fingertapping. (TIF) [file pone.0158185.s002.tif]

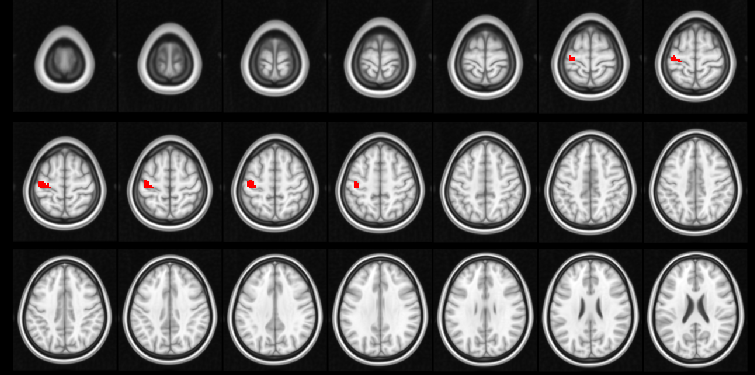

Supplement: S3 Fig — The map shows the region of interest in the right motor area used in Fig 6 of the main manuscript. (TIF) [file pone.0158185.s003.tif]

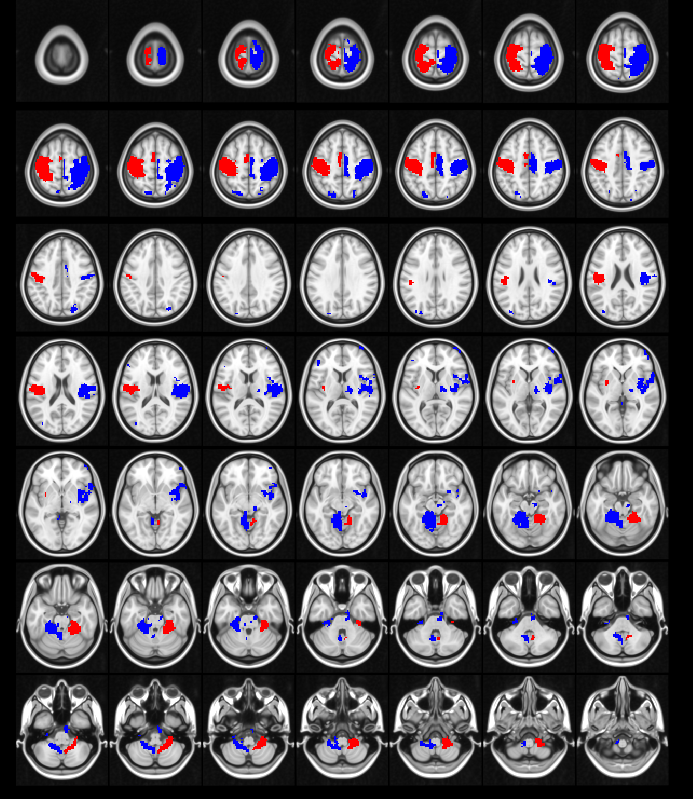

Supplement: S4 Fig — This map is based on the same data as Fig 7 of the main manuscript. (TIF) [file pone.0158185.s004.tif]

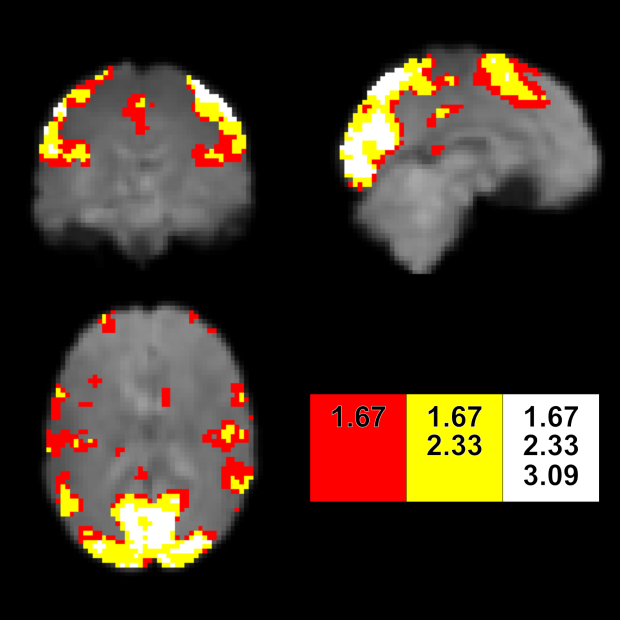

Supplement: S6 Fig — We compared three different initial zt-thresholds for the HCP motor task. The resulting hubness maps were binarized and overlayed over each others for the sake of comparability. The regions displayed in red are only found for the lowest (i.e. most permissive) threshold, regions in yellow are found for both the lowest and middle threshold, and finally regions in white are found in the hubness maps of all thresholds. Note that the hubness maps for the more stringent thresholds did not contain voxels that were undetected in the lower thresholds, that is, the detected regions were always a subset of the next lower threshold. (PNG) [file pone.0158185.s006.png]

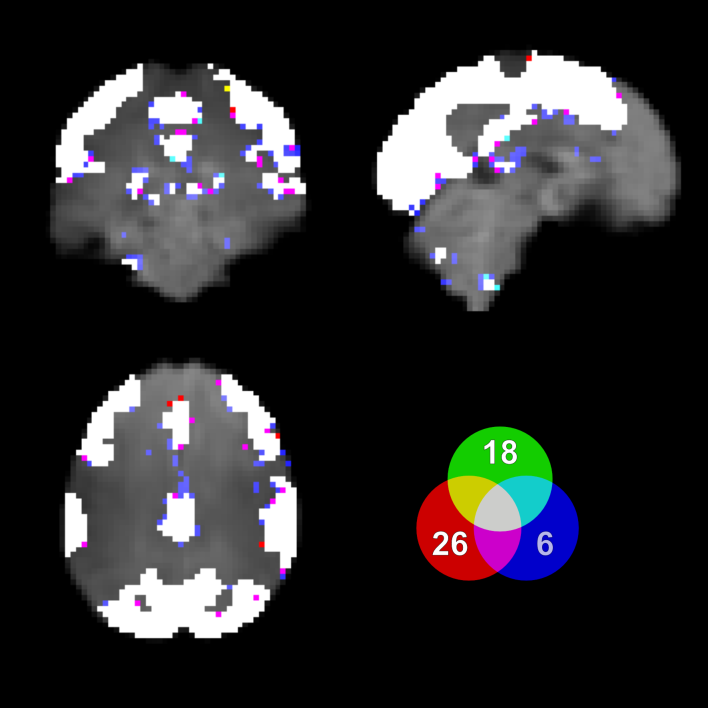

Supplement: S7 Fig — We compared three different definitions of neighborhood adjacency (26, 18 and 6) with each others. We overlayed the results in an additive manner, as in a Venn diagram: voxels in white color are found for all neighborhood schemes, pure colored voxels (red, green and blue) only for one respective neighborhood scheme (26, 18 and 6) and mixed colors (yellow, cyan, magenta) for voxels found in two schemes (26/18, 18/6, 6/26). (PNG) [file pone.0158185.s007.png]

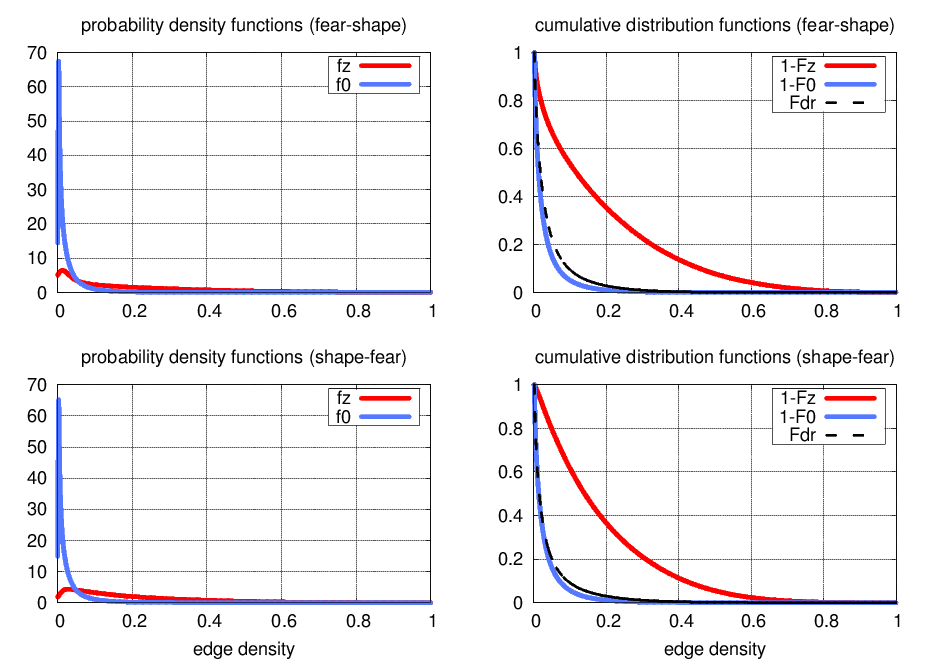

Supplement: S8 Fig — For the “face-shape” contrast, the cutoff was found to be De > 0.1481, i.e. for edges with De > 0.1481 the false discovery rate falls below 0.05. For the reverse contrast, the cutoff was De > 0.1493. The estimation of F0 is based on 1000 random permutations. (PNG) [file pone.0158185.s008.png]

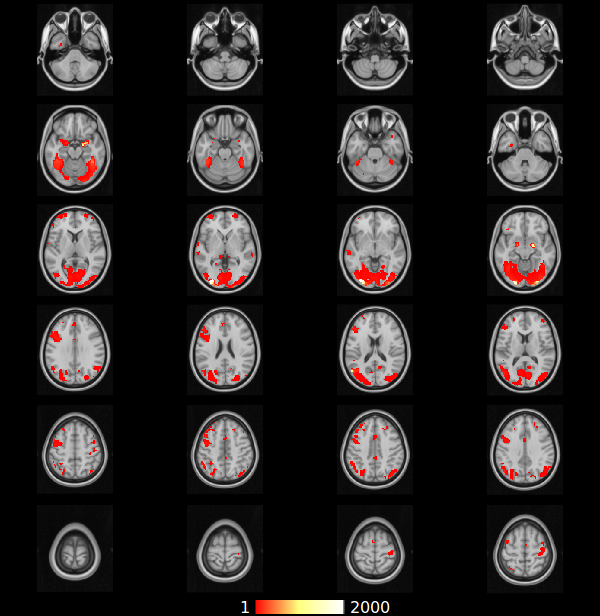

Supplement: S9 Fig — The map is based on TED results thresholded at De > 0.1481 so that fdr < 0.05. It is based on the same data as that of Fig 8 of the main manuscript. (TIF) [file pone.0158185.s009.tif]

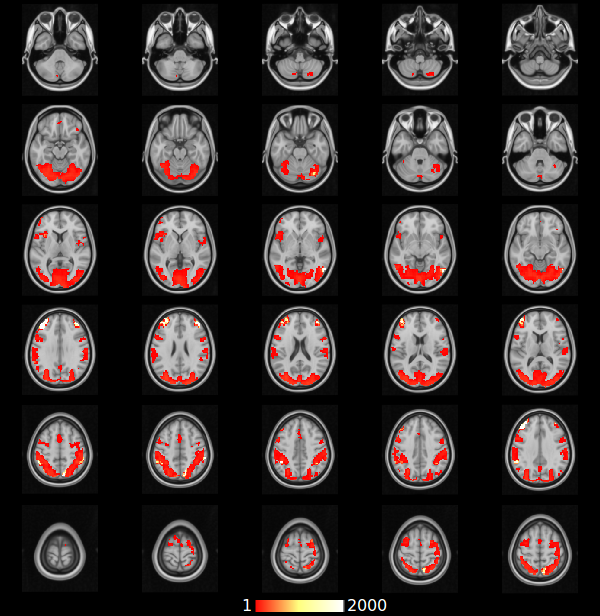

Supplement: S10 Fig — The map is based on TED results thresholded at De > 0.1493 so that fdr < 0.05. It is based on the same data as that of Fig 8 of the main manuscript. (TIF) [file pone.0158185.s010.tif]

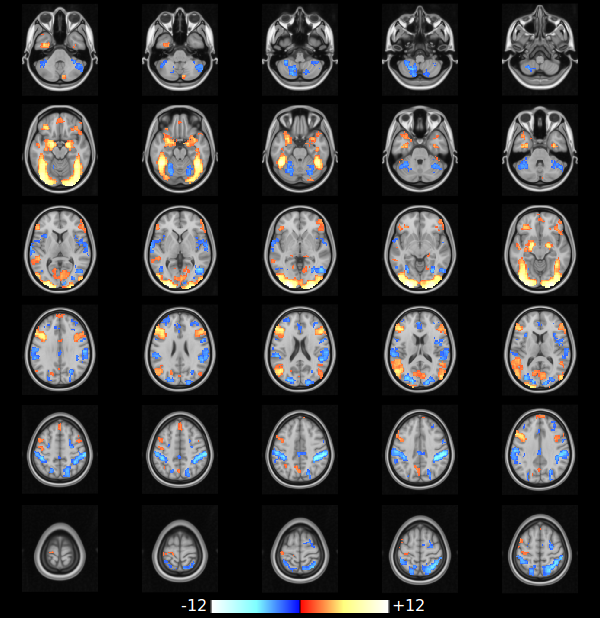

Supplement: S11 Fig — The map shows uncorrected z-values thresholded at |z| > 2.33 following a conjunction of a GLM analysis of the two phase-encoding runs. (TIF) [file pone.0158185.s011.tif]
