# Supplementary material for: Task-Related Edge Density (TED)—A New Method for Revealing Dynamic Network Formation in fMRI Data of the Human Brain
Source: PLoS One. 2016 Jun 24;11(6):e0158185. doi: 10.1371/journal.pone.0158185 (PMC4920409; doi:10.1371/journal.pone.0158185)
Supplement: S5 Fig — The plot shows the distribution of the cutoff-parameter De based in dependency of the number of permutations that are used. The permutations were computed in blocks of 50, which we concatenated to investigate the stability of the cutoff local edge density De. More precisely, we computed the the distribution of De based on 50, 100, 150 and 200 permutations, using 40 random draws of samples each (without replacement). The error bars indicate the standard error across the 40 draws that were performed for each number of permutation. The estimate of De clearly converges for larger number of permutations, as the mean and standard deviation converges after 100 permutations. (PDF) [file pone.0158185.s005.pdf]

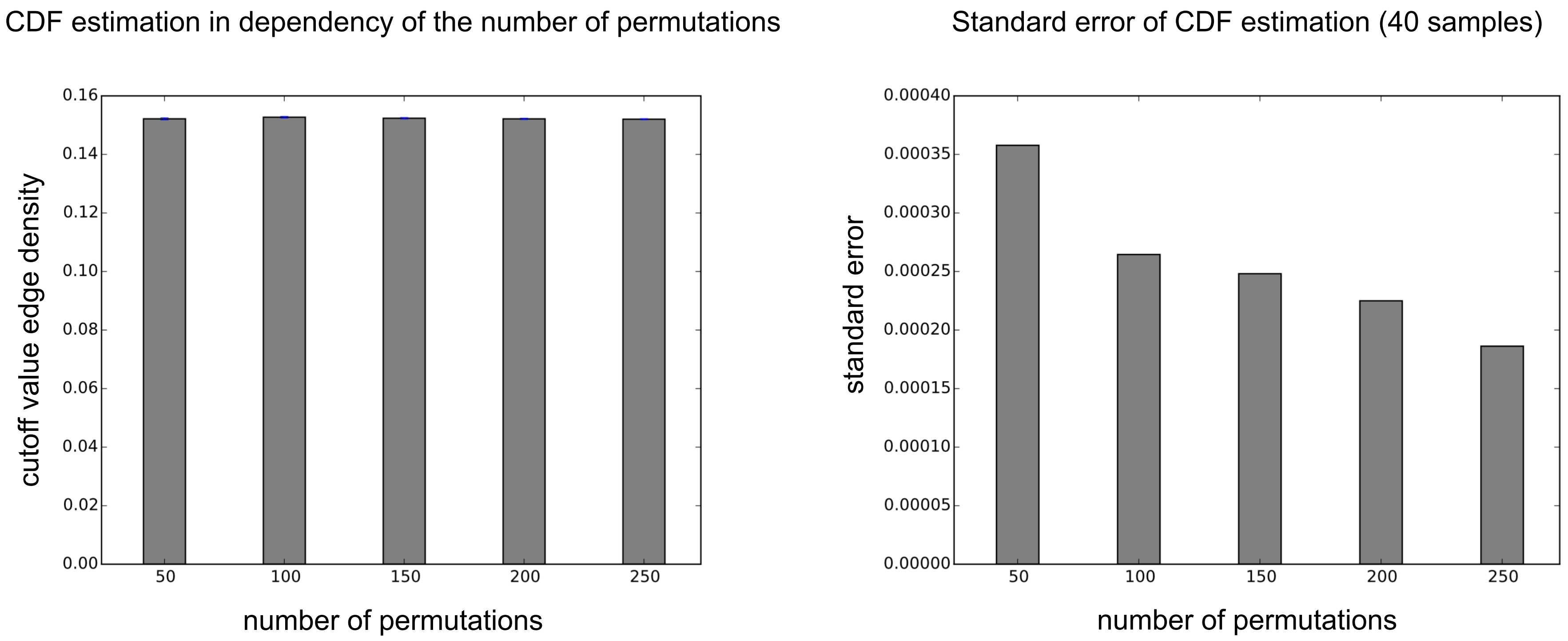
CDF estimation in dependency of the number of permutations
cutoff value edge density
0.16
0.14
0.12
0.10
0.08
0.06
0.04
0.02
0.00
50
100
150
200
250
number of permutations
Standard error of CDF estimation (40 samples)
standard error
0.00040
0.00035
0.00030
0.00025
0.00020
0.00015
0.00010
0.00005
0.00000
50
100
150
200
250
number of permutations
